# Emerging roles of the HECT-type E3 ubiquitin ligases in hematological malignancies

**DOI:** 10.1007/s12672-021-00435-4

**Published:** 2021-10-08

**Authors:** Vincenza Simona Delvecchio, Claudia Fierro, Sara Giovannini, Gerry Melino, Francesca Bernassola

**Affiliations:** Department of Experimental Medicine, TOR, University of Rome “Tor Vergata”, Via Montpellier 1, 00133 Rome, Italy

**Keywords:** Leukemia, Ubiquitin, Ubiquitination, HECT-type E3 ubiquitin protein ligases, Proteasomal degradation

## Abstract

Ubiquitination-mediated proteolysis or regulation of proteins, ultimately executed by E3 ubiquitin ligases, control a wide array of cellular processes, including transcription, cell cycle, autophagy and apoptotic cell death. HECT-type E3 ubiquitin ligases can be distinguished from other subfamilies of E3 ubiquitin ligases because they have a C-terminal HECT domain that directly catalyzes the covalent attachment of ubiquitin to their substrate proteins. Deregulation of HECT-type E3-mediated ubiquitination plays a prominent role in cancer development and chemoresistance. Several members of this subfamily are indeed frequently deregulated in human cancers as a result of genetic mutations and altered expression or activity. HECT-type E3s contribute to tumorigenesis by regulating the ubiquitination rate of substrates that function as either tumour suppressors or oncogenes. While the pathological roles of the HECT family members in solid tumors are quite well established, their contribution to the pathogenesis of hematological malignancies has only recently emerged. This review aims to provide a comprehensive overview of the involvement of the HECT-type E3s in leukemogenesis.

## Introduction

### The ubiquitin system

Ubiquitination is an essential posttranslational modification that involves the covalent attachment of the conserved 76-amino acid polypeptide ubiquitin to a target protein. The ATP-dependent and reversible reaction is a multistep process mediated by three classes of enzymes: E1 activating enzymes (E1), E2 conjugating enzymes (E2) and E3 ubiquitin proteins ligases (E3) [1–3]. In the first reaction, following ATP-dependent activation of ubiquitin, there is the formation of a thioester linkage between ATP-ubiquitin and the catalytic cysteine residue of the E1. Ubiquitin is then transferred to the E2 enzyme via a similar thioester linkage. In the final step of the ubiquitination enzymatic cascade, the E3s coordinate the transfer of ubiquitin from the E2 to a substrate or can directly promote the attachment of ubiquitin to the target polypeptide. Ubiquitin is transferred to the e-amino group of a lysine residue or to the amino-terminus of a polypeptide. The reverse reaction is carried out by deubiquitinases, proteases that remove ubiquitin chains from proteins and then release free ubiquitin from these polymers [4, 5].

The ubiquitination process can involve either the attachment of a single ubiquitin molecule (monoubiquitination) or the formation of a ubiquitin chain on a target protein (polyubiquitination). To form polyubiquitin chains, ubiquitins are linked via different internal lysine residues (K6, K11, K27, K29, K33, K48, K63) [6]. In addition, polyubiquitin chains can be generated through peptide bond formation between the C-terminal glycine of one ubiquitin and the amino-terminal alpha-amino group of the N-terminal methionine (M1) residue of the next ubiquitin [7–9]. Polyubiquitin chains linked through different lysine residues of the ubiquitin molecule can adopt different structures, dictating distinct fates of the modified proteins [6, 10]. Thus, ubiquitination can have different functional consequences, depending on the residue of the ubiquitin that is employed to form the chain. Protein modification by ubiquitin can result in proteasomal degradation of the target polypeptide, influence the activity, the subcellular localization and the interactions of proteins or promote the formation of signaling complexes [10]. In particular, K48-linked ubiquitin chains target proteins for proteolytic degradation by the 26S proteasome [11]. The ubiquitin proteasome system (UPS) is composed of the E1, E2 and E3 ubiquitin enzymes and the 26S proteasome.

Modification of proteins by ubiquitin is involved in the regulation of various relevant biological processes, including apoptosis, cell cycle progression, differentiation, transcription, signal transduction, DNA repair and autophagy [12–19]. Coherently, dysregulation of the UPS is associated with severe pathologies, such as neurodegenerative and immune disorders and cancer [20–26]. Indeed, the autophagic process, reviewed in [27–30], strongly relies on the protein degradation pathway.

### HECT E3 protein ubiquitin ligases

**Fig. 1:**
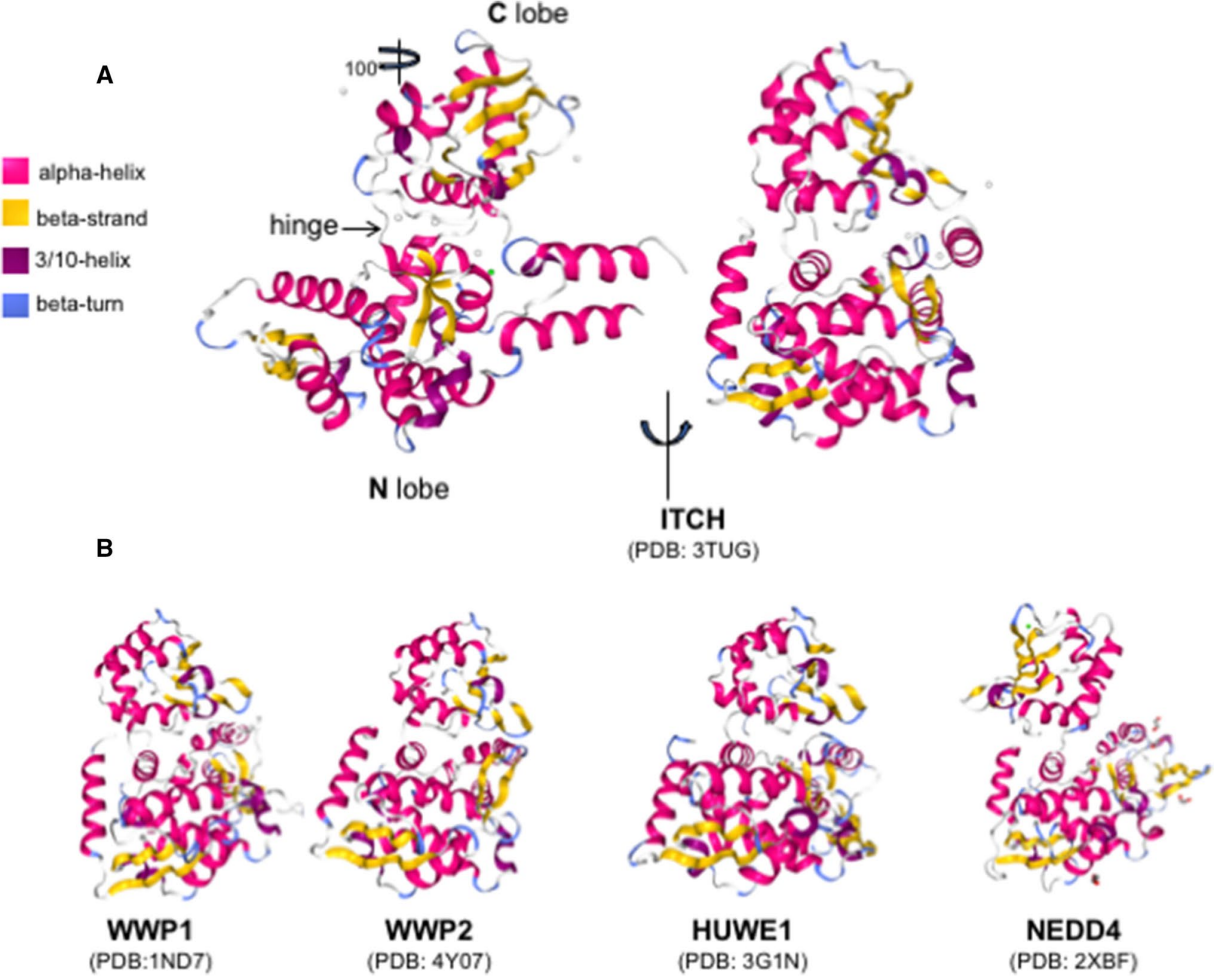
**A** Structural models of ITCH (PDB: 3TUG) [114] showing the two lobes, with the hinge region in two orthogonal views. **B** Comparison of the HECT structure among different members of the sub-family, to highlight the high structural similarity between WWP1 (PDB: 1ND7) [33], WWP2 (PDB: 4Y07) [115], HUWE1 (PDB: 3G1N) [116], and NEDD4 (PDB: 2XBF) [117]

Homologous to E6AP C-Terminus (HECT)-type enzymes are a subfamily of E3s that comprises 28 members [31, 32]. HECT E3s directly catalyze substrate ubiquitination in a two-step process. They first load activated ubiquitin from the E2 on themselves, through a ubiquitin-thioester catalytic intermediate with a catalytic cysteine located in the HECT domain; then, they transfer ubiquitin to the substrate. The HECT domain is composed of two lobes connected by a short unstructured linker: the N-terminal lobe containing the E2 binding site, and the smaller C-terminal lobe including the catalytic cysteine (Fig. 1). The flexibility of the linker region is required to juxtapose the catalytic residues of the E2 and E3 [33].

**Fig. 2:**
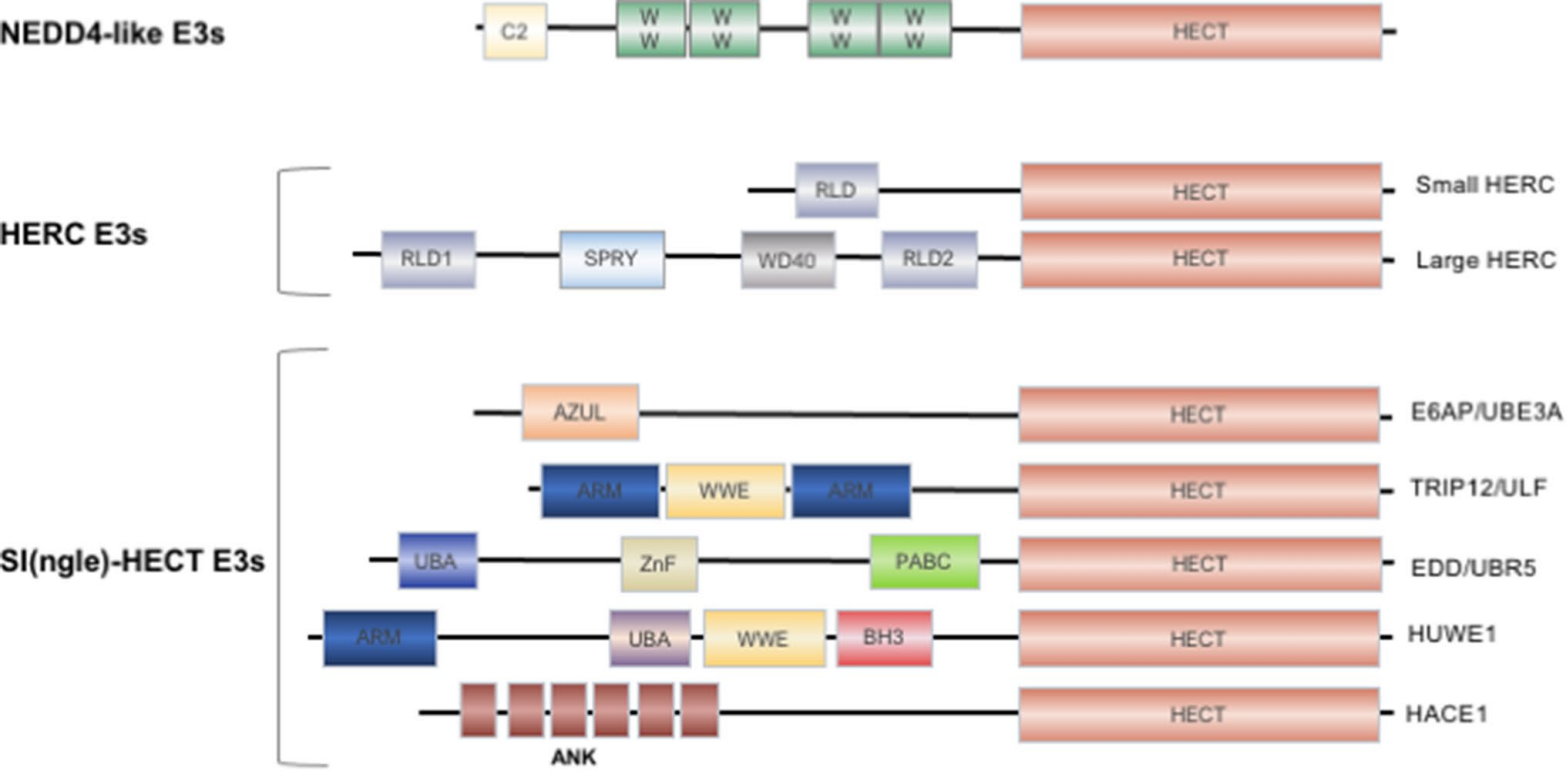
Structural features of HECT‐type E3 enzymes. All the family members share the presence of the catalytic HECT domain, which is located at the C-terminus. According to their N‐terminal protein–protein interaction domains, the HECT E3s have been divided into three subgroups. The NEDD4-like members contain an N‐terminal protein kinase C‐related C2 domain and two‐four central WW domains that mediate substrate recruitment. HERC E3s possess a single (small HERCs) or more (large HERCs) RCC [regulator of chromatin condensation 1]-like domain (RLDs) preceding the HECT domain. Large HERCs contain additional domains, such as SPRY and WD40 domains. The SI(ngle)‐HECT subfamily is composed of varied number and types of domains such as armadillo repeat‐containing domain (ARM), amino‐terminal Zn‐finger of Ube3a ligase domain (AZUL), WWE domain (WWE), Bcl-2 homology 3 domain (BH3), ankyrin repeat‐containing domain (ANK), polyadenylate‐binding protein C‐terminal domain (PABC), ubiquitin-associated domain (UBA), and zinc finger domain (ZnF)

HECT E3s can either directly recruit their targets through specific protein–protein interaction modules located within their N-terminal region or require accessory or adaptor proteins to recognize the substrate [34]. HECT-type E3s assemble poly-ubiquitin chains by employing all the ubiquitin linkage types, with each family member displaying different chain type specificity [35, 36]. Based on the distinct structural features of the N-terminal protein–protein interaction modules, the HECT E3s have been further classified into three sub-families: the NEDD4-like (also referred as the C2-WW-HECT), the HERCs and the “Other”-HECT E3s (Fig. 2). The NEDD4-like enzymes share a modular architecture that consists of an N-terminal protein kinase C-related C2 domain, and two to four tryptophan-tryptophan (WW) protein interacting domains, preceding the HECT domain (Fig. 2). In humans, this subfamily is composed of nine members: NEDD4-1, NEDD4-2, ITCH, SMURF1, SMURF2, WWP1, WWP2, NEDL1, and NEDL2 [37]. WW domains are protein modules that mediate protein–protein interactions through the recognition of proline-rich peptide motifs and phosphorylated serine/threonine-proline sites. The six members of the HERC sub-family of E3s bind substrates through one or more regulator of chromosome condensation 1 (RCC1)-like domains (RLDs) (Fig. 2). They are further classified into small and large HERCs, containing a single or multiple RLDs, respectively. The third subfamily includes enzymes that contain neither WW nor RLDs domains. They recruit substrates through a variety of substrate-binding modules located at their N-terminus, with many members having more than one domain (Fig. 2).

Aberrant expression, mutations, or deregulated activity of the HECT enzymes have been associated with tumorigenesis [31, 32]. HECT E3s can contribute to tumour development by controlling the ubiquitination of substrates that function as either tumour suppressors or oncogenes. The pathological roles of the HECT family members in hematological malignancies, particularly in myeloid related neoplasms, have only recently emerged. In this review, we will highlight the contribution of the HECT-type E3s in leukemogenesis.

### Dysregulation of the UPS in hematopoietic malignancies

Leukemias are a group of life-threatening malignant disorders of the blood and bone marrow characterized by extensive biologic diversity. They are classified according to the type of blood cells affected, the morphology and immunophenotype of the leukemic blasts, and how quickly the disease progresses (Table 1). The dominant leukemia cells can be either mature cells, such as in chronic lymphocytic leukemia (CLL) or precursor (stem/progenitor) cells of various lineages, such as in the acute leukemias, or both precursor and mature cells, such as in chronic myeloid leukemia (CML). The UPS is the main cellular degradation machinery that regulates the turnover of crucial proteins involved in tumorigenesis. Dysregulation of the UPS can therefore contribute to the initiation and progression of neoplastic transformation. Three common types of alterations in the UPS may occur in hematopoietic malignancies: (1) mutations in E3s leading to impaired ubiquitylation of their own substrates [38, 39], (2) altered expression of E3s (overexpression/deletion) resulting in the aberrant degradation or accumulation of their targets [40–42], and (3) mutations in substrates that impair their recruitment by E3s [43]. Several evidences strongly link leukemogenesis to pathways where proteostasis is essential, like for example NRF2 and IAP [44–47], and where BH3 profiling may offer therapeutic venues [48–51].

**Table 1:** Type of transformed cell and molecular pathways involved in leukemia pathogenesis

Hematological disorders	Features	Molecular/chromosomal alterations	Global incidence (cases/ 100,000 inhabitants/year)	HECT E3s implicated in leukemia pathogenesis/progression
Acute myeloid leukemia (AML)	A genetically very heterogeneous disorder characterized by the accumulation of acquired somatic mutations, epigenetic changes, chromosomal aberrations in hematopoietic progenitor cells that alter their self-renewal, proliferation, and differentiation	Heterogeneity of molecular defects. Frequent somatic mutations include: NPM1, FLT3 and CEBPA, RUNX1, Somatic mutations in epigenetic modifiers include: DNMT3A, IDH1/2 and TET2 Cytogenetic abnormalities include: t(8;21), t(15;17), t(9;22) and inv(16)	3,5	WWP1 [42], HERC1 [94], E6AP [98, 99]
Chronic myelogenous leukemia (CML)	Clonal myeloproliferative disease characterized by leukocytosis and an accumulation of granulocytes and their precursors	The hallmark of CML is the (9;22)(q34;q11) reciprocal chromosomal translocation, leading to the constitutive expression of the fusion oncoprotein BCR–ABL	1,5	SMURF1 [56], HERC1 [94],
Acute lymphoblastic leukemia (ALL)	An aggressive hematological tumor, driven by malignant transformation and expansion of T-cell progenitors. It is the most common type of leukemia in children. ALL accounts for 74% of pediatric leukemia cases	One of the hallmarks of the disease is the Philadelphia chromosome positivity as a result of the t(9;22)(q34;q11). that gives rise to the *BCR‐ABL* oncogene. Philadelphia negative ALL are subcategorized on the basis of different classes of genetic alterations: type I, ABL‐class fusions; type II, erythropoietin‐receptor or *JAK2* rearrangements; type III, cytokine receptor‐like factor 2 rearrangements; type IV, mutations activating JAK‐STAT signaling; type V, uncommon kinase mutations; type VI, RAS‐pathway mutations; type VII, no mutations in kinase genes	1,8	HERC1 [90–92]
Chronic lymphocytic leukemia (CLL)	A mature B cell neoplasm characterized by a progressive accumulation of monoclonal B lymphocytes	More than forty mutated driver genes have been identified including NOTCH, FBXW7, KRAS, p53 and ATM Approximately 80% of CLL patients carry at least 1 of 4 common chromosomal alterations: del13q14, del11q22-23, del17p12, and trisomy 12	4,9	ITCH [79], NEDD4 [86]
Myeloproliferative neoplasms (MPNs)	Heterogenous group of acquired clonal hematopoietic stem cell disorders characterized by an abnormal proliferation of myeloid cells. MPNs include polycythemia vera, essential thrombocythemia, and primary (idiopathic) myelofibrosis. MPN patients can spontaneously transform into either myelodysplastic syndrome or AML	Somatic mutations (JAK2, calreticulin, thrombopoietin receptor	2,17	HERC1 [94]

### Regulation of leukemic oncoproteins turnover by the UPS

Chromosome translocations can lead to the expression of chimeric proteins that act as oncogenic drivers in leukemia [52]. Deregulated protein stability, as a result of chromosomal translocations, can be a driving pathogenic factor in leukemias. Over 95% of cases of CML and about 15% of acute lymphocytic leukemia (ALL) are associated with the Philadelphia chromosome. This genetic abnormality results from the (9;22)(q34;q11) reciprocal chromosomal translocation that creates the constitutively active tyrosine kinase BCR (breakpoint cluster region)-ABL (Abelson proto-oncogene) fusion protein. BCR-ABL activates several oncogenic signalling pathways leading to neoplastic transformation, tumor growth and proliferation. The tyrosine kinase activity of BCR-ABL largely derives from the ABL tyrosine kinase. BCR-ABL is the ideal therapeutic target for CML. Indeed, imatinib, a potent and selective inhibitor of ABL kinase activity, is widely used for CML treatment. However, disease progression is associated with decreased responsiveness to imatinib, due to amplification or point mutations in the BCR-ABL gene. BCR-ABL-dependent resistance remains a major challenge in the field, and novel strategies are still required in CML therapy. The stability of the BCR-ABL protein is regulated by the ubiquitin–proteasome pathway [53]. A number of E3s, including CHIP, c-CBL, and SH2-U-box have been involved in BCR-ABL polyubiquitination and subsequent degradation [54, 55]. Importantly, ABL undergoes protein degradation significantly faster than the BCR-ABL oncoprotein [56], indicating that a different regulation of ABL and BCR-ABL protein stability may contribute to BCR-ABL–mediated tumorigenesis.

Degradation of fusion oncoproteins can also offer a therapeutic strategy for leukemia eradication, as for acute promyelocytic leukemia (APL), a clinical subtype of acute myeloid leukemia (AML). This leukemia comprises a clinically heterogeneous group of hematological malignancies, with varying karyotypic, genetic and epigenetic abnormalities. The disease is characterized by uncontrolled proliferation and altered differentiation of myeloid progenitor/stem cells. APL is curable by using a combination of all-trans-retinoic acid (ATRA) and arsenic trioxide (ATO) that restores the ability of leukemic cells to differentiate [57]. Mechanistically, this treatment triggers the disposal of the promyelocytic leukemia protein (PML)/retinoic acid receptor-alpha (RARalpha) chimeric oncoprotein, which results from the t(15;17) chromosomal translocation and is responsible for the differentiation block of APL blasts. PML-RARalpha binds to RA responsive elements on DNA with high affinity and recruits transcriptional repressive complexes that inhibit the expression of RARalpha target genes necessary for myeloid differentiation. ATRA and ATO induce degradation of PML-RARalpha through several cooperating proteolytic mechanisms including caspase-3-mediated cleavage, proteasomal degradation and autophagy [58]. Similarly, elimination of the BCR-ABL fusion protein through autophagy has been shown in CML, where imatinib has the dual effects of inhibiting the tyrosine kinase activity of BCR-ABL, and inducing its degradation [59].

More broadly, prognosis of patients with AML is strongly influenced by the type of chromosomal or genetic alteration, as well by changes in gene expression. Approximately one-third of AML patients do not achieve complete remission in response to chemotherapy and, even when complete remission is achieved, about 70% of patients relapse within 5 years. Thus, despite great progress in defining molecular determinants involved in AML pathogenesis, much of the variability of the alterations characterising this haematological disorder is still a relevant open question in the field, and there is urgent need to identify novel therapeutic targets.

## Role of the HECT E3s in the pathogenesis of leukemia

### WW domain-containing E3 ubiquitin protein ligase 1 (WWP1)

**Fig. 3:**
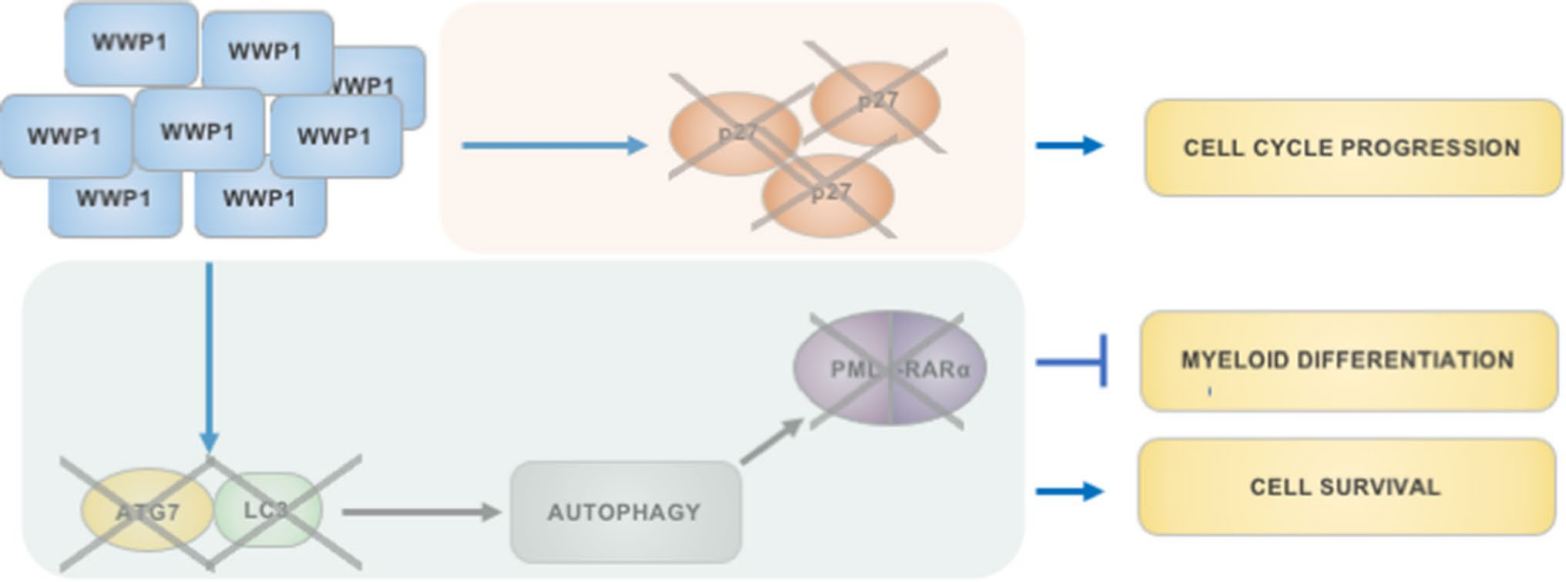
A model for the outcome of WWP1 overexpression in AML blasts. Elevated amounts of WWP1 may limit the stability of the cell cycle inhibitor p27^Kip^, thus promoting cell cycle progression and proliferation. WWP1 can also interfere with the basal autophagic flux, possibly by negatively regulating ATG7 and LC3 levels, hence preventing autophagy and increasing blast survival. In leukemias bearing oncoproteins (e.g. (PML-RARalpha) susceptible to autophagy-mediated disposal, WWP1 overexpression would counteract their proteolysis. As a consequence, WWP1 would prevent myeloid differentiation

WWP1 (WW domain-containing E3 ubiquitin protein ligase 1) possesses a C2 N-terminal domain, followed by four WW domains that precede the HECT domain. The C2 domain mediates WWP1 localization at cellular membranes. Indeed, WWP1 is predominantly localized to plasma membrane, endosomes and Golgi apparatus, though it is also partially nuclear. Its expression is frequently deregulated in tumors, in which WWP1 plays an oncogenic role as a positive modulator of cancer cell proliferation and survival [42, 60–62]. High expression of WWP1 is generally associated with poor prognosis. In AML, WWP1 overexpression confers a proliferative advantage to leukemic blasts, by limiting the stability of p27^Kip^ and, ultimately, promoting cell cycle entry [42] (Fig. 3). In addition, WWP1 overexpression represses basal autophagy thus increasing cell viability and inhibiting differentiation of leukemic blasts. In particular, in APL cells, re-establishment of the autophagic flux via WWP1 silencing is responsible for myeloid maturation of leukemic blasts expressing oncoproteins that are sensitive to autophagy-dependent proteolysis (*e.g.* PML-RARalpha and FLT3/ITD) (Fig. 3). Autophagy-dependent destabilization of PML-RARalpha upon WWP1 inactivation results in a partial restoration of ATRA target-gene expression, including genes involved in granulocytic differentiation. From a molecular perspective, WWP1 seems to prevent autophagy activation by affecting ATG7 and LC3 stability, thus interfering with the formation and elongation of autophagosomes. Though still speculative, data from Sanarico and colleagues [42] suggest that WWP1 would favor the degradation of autophagic proteins that are involved in the elongation and closure of the autophagosomal membranes, thus ultimately inhibiting the autophagic flux. The prevalent subcellular localization of WWP1 to membrane compartments, which represent the relevant nucleation sites for autophagosome formation, further implies WWP1 as a regulator of the early steps of autophagy.

### ITCH

ITCH was initially identified through genetic studies examining the mutation of the *agouti* locus, which causes coat-colour alterations and itching of the skin in mice [63, 64]. The expression of both *agouti* and *itch* genes is affected by the 18H mutation, which characterizes *Itchy* mice with severe immune and inflammatory defects [64, 65]. ITCH has been additionally shown to be required for the development of immune cells. In particular, it regulates the differentiation of T lymphocytes via its Jun family protein substrates, c-Jun and JunB [66].

**Fig. 4:**
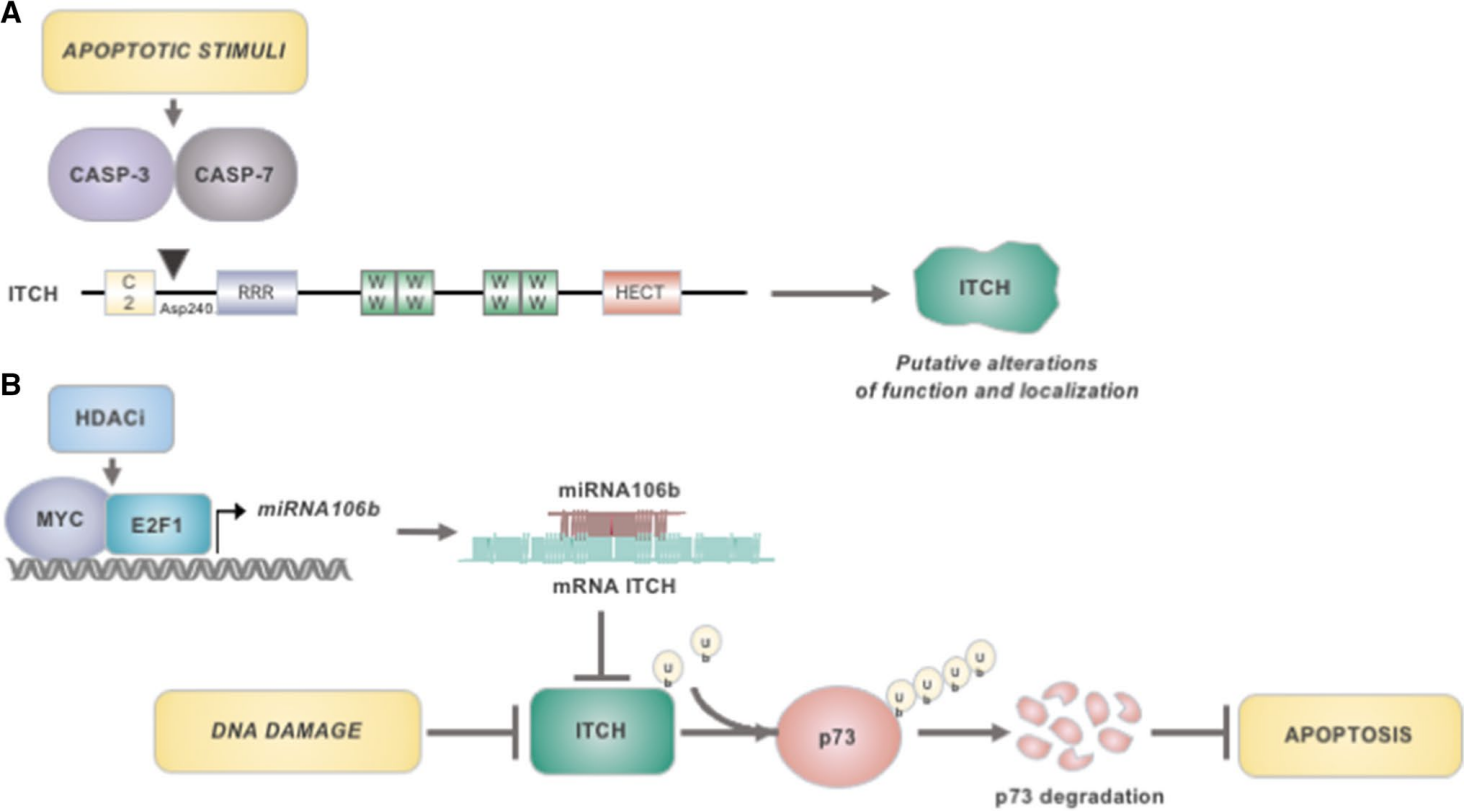
Implications of ITCH in apoptotic cell death of CLL cells. **A** Upon apoptotic stimuli*,* Caspase-3 and Caspase-7-mediated cleavage of ITCH on Asp240 residue may affect its function and localization by releasing an N-terminal fragment that contains the JNK1 regulatory domain and the C2 domain, respectively. **B** Both DNA damage (e.g. chemotherapeutic drugs) and HDAC inhibitors promote ITCH downregulation. HDAC inhibition activates E2F1- and Myc-mediated transcription of miR106b, promotes ITCH downregulation. ITCH reduces p73 levels by targeting it for poly-ubiquitination and proteasomal degradation, thus inhibiting apoptosis

ITCH is interestingly emerging to have a role in tumorigenesis, and in haematological malignancies as well. Most of its binding partners and targets are indeed involved in signalling, differentiation and apoptosis [66–69]. ITCH itself is a substrate of apoptotic effectors, such as caspases, which cleave it in CLL primary samples [70] (Fig. 4). The enzymatic cleavage (Asp240) causes the loss of an N-terminal ITCH fragment that contains a JNK1 regulatory region [71, 72]. Phosphorylation of this regulatory domain by JNK1 is necessary for disrupting an intramolecular inhibitory interaction, and to induce a conformational change that enhances the catalytic activity of ITCH [72]. The cleavage by caspases might therefore interfere with ITCH ubiquitination function. In addition, since the N-terminal C2 domain of the NEDD4-like E3s is important for their subcellular localization [73], the caspase activity on ITCH may affect it as well [70].

ITCH involvement in cell death regulation is further demonstrated by the crucial role of one of its substrates, the tumour suppressor p73. The latter is rarely mutated in cancer [74, 75], differently from p53, which instead accumulates mutations also in CLL [76]. p73 efficiently binds p53 responsive elements for transcription activation of proapoptotic target genes [77]. Under basal conditions, ITCH maintains p73 at low levels, by binding the tumor suppressor via its C-terminal PPPY motif and targeting it for polyubiquitination and subsequent proteasomal degradation [68]. In response to genotoxic stress, ITCH expression is downregulated, leading to the stabilization and concomitant transcriptional activation of p73 (Fig. 4).

A similar mechanism has been described in CLL, a disorder characterized by dysfunctional apoptosis and low miRNAs expression, which are thought to be responsible for the resistance of leukemic cells to cell death [78]. Sampath and collaborators [79] have reported that treatment of CLL primary cells with deacetylase inhibitors reactivates E2F1- and MYC-mediated transcription of miR106b, which, in turn, promotes ITCH downregulation and p73 stabilization (Fig. 4). As a result, p73-mediated PUMA transcription is induced, leading to mitochondrial dysfunction, activation of caspases and apoptosis of CLL cells [79]. Such body of evidence suggests that the use of deacetylase inhibitors for ITCH inhibition may be considered as a novel antitumor approach for CLL therapy.

### Neuronal precursor cell-expressed developmentally down-regulated (NEDD4)

NEDD4, the prototypic member of the NEDD4-like subgroup, plays a role in different cellular functions including inflammation, adaptive immunity, proliferation, apoptosis and carcinogenesis [80]. Abnormal expression of NEDD4, has been reported in several human cancers including, prostate, bladder and colon carcinomas [81], and its overexpression is frequently associated with disease progression [82]. However, dual roles for NEDD4 in carcinogenesis have been described. The majority of the studies converge towards an oncogenic role for NEDD4. Its oncogenic functions are mainly the result of its ability to activate the PI3K/AKT signalling pathway [83, 84]. Mono-ubiquitination of PTEN by NEDD4, instead, appears to have a tumour suppressive outcome, because it drives PTEN cytoplasmic/nuclear shuttling [85].

The extent of NEDD4 contribution to haematological malignancies is still far to be understood. Shukla et al. [86] have investigated the expression of NEDD4 in CLL cells and focused on its possible upstream regulatory factors. Characterization of Interferon Regulatory Factor (IRF4) deficient mice that develop spontaneous CLL revealed a dramatic reduction of NEDD4 and consequent hyperactivation of the NOTCH signaling, which is indispensable for leukemia development. Indeed, abnormal activation of the NOTCH signaling pathway is one of the most recurrent molecular alterations in human CLL [87]. Being a transcriptional target of IRF4 [86] and acting as an E3 for NOTCH [88], NEDD4 provides a functional link between IRF4 and the NOTCH signaling pathway in CLL cells.

### SMAD-specific E3 ubiquitin protein ligase 1 (SMURF1)

SMURF1 acts as an E3 for ABL that is rapidly degraded by the proteasome upon ubiquitination [56, 89]. ABL modification by SMURF1 is dependent on the first 45 amino acids of the kinase that, as a result of the chromosomal translocation, are not present in the BCR-ABL chimera [56]. This N-terminal domain of ABL contains a degradation signal that is required for its interaction with SMURF1 and contains four lysine residues, whose modification regulates ABL turnover. Coherently, both BCR-ABL and a deletion mutant of ABL (ABL^D45^), lacking the first 45 amino acids, but not ABL, drive growth factor-independent proliferation and survival of the human erythroleukemic cell line TF-1 [56]. Similarly, ABL^D45^ expression promotes tumor development and growth in xenotrasplantation models [56]. In conclusion, the study by Yan and colleagues [56] demonstrates that loss of the N-terminal ABL domain, caused by the (9;22)(q34;q11) chromosomal translocation, prevents ABL degradation thus contributing to the oncogenic potential of the BCR-ABL fusion protein.

### HERC1

HERC1 is one of the large HERC enzymes, whose contribution to tumorigenesis has only recently began to be explored. Accumulating evidences have highlighted a role for HERC1 as a tumor suppressor. Point mutations in HERC1 have been detected in AML, T-ALL and T-cell prolymphocytic leukemia [90, 91]. Somatic deletions of HERC1 have been observed in newly diagnosed ALL, in which they have been associated to an increased resistance to antileukemic agents [92]. These leukemias express high mRNA levels of the DNA mismatch repair enzyme MSH2, while its protein levels are low or undetectable, suggesting the existence of a post-transcriptional regulatory mechanism. HERC1 functions as a positive regulator of MSH2 protein stability; thus, reduction of MSH2 protein amount and chemoresistance are possibly achieved as a result of HERC1 deletion/inactivation in ALL cells. In addition, though very rare, an atypical HERC1-PML transcript fusion mRNA has been described in APL, though the functional outcome of this chimera has not been yet investigated [93]. Ali and collaborators [94] have recently reported that HERC1 expression is dysregulated in myeloid related neoplasms. At diagnosis, HERC1 transcript levels are markedly down-regulated in AML, CML and primary myelofibrosis, an aggressive form of myeloproliferative neoplasm (MPN), relatively to bone marrow and peripheral blood control cells. In particular, in AML patients, HERC1 deregulation was not found associated with any molecular or chromosomal alteration. In CML patients, molecular remission was associated with restoration of HERC1 expression to the levels of control subjects, while, at relapse, the amount of the E3 underwent a further decline. At the molecular level, HERC1 is a protein interactor and a substrate for BCR-ABL1-mediated phosphorylation. To date, whether HERC1 may act as a BCR-ABL1 negative modulator, or if its phosphorylation by BCR-ABL1 may regulate its enzymatic activity, as for other family members, remain open questions. Similarly, the specific contribution of HERC1 to normal hematopoiesis and leukemogenesis as well as the identification of the involved substrates require further investigation.

### E6-associated protein (E6AP)

E6AP is a member of the “Other”-HECT E3 sub-group and was originally identified as a protein involved in the human papillomavirus E6-oncoprotein-induced degradation of p53 [95]. However, a number of E6-independent targets of E6AP have been identified [32]. Amongst these, PML has been reported as a target for E6AP-mediated ubiquitin/proteasomal degradation in normal lymphoid cells [96] and in B-cell lymphomas [97]. The E6AP-PML axis plays a role in B-cell lymphomagenesis. E6AP is overexpressed in Burkitt lymphoma tumor cells, in which it counteracts the ability of PML to induce cellular senescence [97].

Another relevant target of E6AP in leukemogenesis is C/EBPα [98], a transcription factor that regulates myeloid differentiation and is mutated or deregulated in AML. E6AP-dependent disposal of C/EBPα negatively affects its transcriptional activity. By targeting C/EBPα for degradation, E6AP contributes to inhibit granulopoiesis of AML cells. Furthermore, E6AP prevents myeloid maturation inducing the proteolysis of MAX-binding protein MNT [99]. MNT is a member of the MYC/MAX/MAD network of transcription factors that acts as a MYC antagonist. It is up-regulated in response to differentiating agents and is a key mediator of ATRA-induced myeloid growth arrest and granulocytic differentiation [99]. Kapoor and colleagues [99] reported that ATRA rescues MNT from proteasomal degradation by inhibiting E6AP. Although further studies are needed to unveil the molecular basis of E6AP inhibition by differentiating agents, the Authors suggested a potential auto-degradation mechanism for E6AP. Altogether, these studies indicate that targeting E6-AP may offer a therapeutic strategy to restore myeloid differentiation of AML blasts.

## Conclusions

In brief, the work on the HECT E3s expands our understanding of the pathogenesis of hematological diseases, providing novel effectors, whose biological significance and therapeutic interest might be explored in the future, especially considering the development of precision oncology [100–102]. This field has opened several novel opportunities, thanks to new algorithms [103–105] and artificial intelligence [106], modernizing the field of precision oncology in general [107–109] as well as in specific cancers [110–113]. From this overview, it appears clear that further studies are needed to corroborate the implications of HECT-type E3s in leukemogenesis, to understand the molecular basis of their oncogenic function in hematological disorders, as well as to identify their relevant substrates and regulators. A remarkable challenge in the field is to establish whether HECT E3s can serve as targets for therapeutic intervention along with the identification of inhibitors for clinical application. Considerable effort has been made in the generation of HECT inhibitors. However, since the catalytic domain is highly conserved amongst the family members and, at least for the NEDD4-like sub-group, the E3s share an elevated degree of protein similarity, selective inhibitors have not yet been identified.

## Data Availability

Not applicable.

## Abbreviations

E1: E1 activating enzyme,
E2: E2 conjugating enzyme,
E3: E3 ubiquitin protein ligases,
UPS: Ubiquitin proteasome system,
HECT: Homologous to E6AP C-Terminus,
RLDs: RCC [regulator of chromatin condensation 1]-like domain,
CLL: Chronic myelogenous leukemia,
T-ALL: T-acute lymphocytic leukemia,
BCR-ABL: Breakpoint cluster region-Abelson proto-oncogene
APL: Acute promyelocytic leukemia,
ALM: Acute myeloid leukemia,
ATRA: All-trans-retinoic acid,
ATO: Arsenic trioxide,
PML: Promyelocytic leukemia protein,
RARalpha: Retinoic acid receptor-alpha
WWP1: WW domain-containing E3 ubiquitin protein ligase 1,
NEDD4: Neuronal precursor cell-expressed developmentally down-regulated,
IRF4: Interferon Regulatory Factor 4,
SMURF1: SMAD-specific E3 ubiquitin protein ligase 1,
E6AP: E6-associated protein,
ARM: Armadillo repeat‐containing domain,
AZUL: Amino‐terminal Zn‐finger of Ube3a ligase domain,
WWE: WWE domain,
BH3: Bcl-2 homology 3 domain,
ANK: Ankyrin repeat‐containing domain,
PABC: Polyadenylate‐binding protein C‐terminal domain,
UBA: Ubiquitin-associated domain,
ZnF: Zinc finger domain
